# Supplementary material for: Unveiling Aducanumab’s safety profile: A comprehensive pharmacovigilance analysis
Source: PLoS One. 2025 Jul 14;20(7):e0328076. doi: 10.1371/journal.pone.0328076 (PMC12258566; doi:10.1371/journal.pone.0328076)
Supplement: Attachment — Submitted filename: Response to Reviewers.docx [file pone.0328076.s002.docx]

Response to Reviewers

We thank the editor and the reviewers for their valuable comments and suggestions, which have greatly improved the quality and clarity of our manuscript. We have revised the manuscript accordingly and provide detailed responses below.

Reviewer 1:

Comment 1:

The authors have not discussed whether the incidence or severity of adverse events of Aducanumab varies by stage of Alzheimer’s disease, such as in patients with MCI versus those with moderate to severe AD.

Response:

We appreciate this insightful comment. However, we would like to clarify that the FAERS database does not include detailed clinical information such as Alzheimer's disease staging (e.g., MCI, mild, moderate, or severe). Therefore, it is not possible to stratify the safety signals of Aducanumab by disease stage using this data source. We have now explicitly acknowledged this limitation in the Discussion section and emphasized that future studies utilizing clinical registries or electronic health records with disease severity information are needed to explore this important aspect.

Comment 2:

The authors have not reported any significant adverse drug interactions between Aducanumab and other medications. Are there known or potential drug-drug interactions?

Response:

We appreciate this point. We reviewed the FAERS data and found no consistent or significant signals suggesting specific drug-drug interactions. We have added clarification to the Results section (section 3.1) stating that no reliable signals for drug-drug interactions were identified.

Reviewer 2:

Comment 1: Causality Limitations Not Adequately Emphasized

Authors sometimes write as if causal relationships are established when using FAERS data, which can only show associations.

Response:

Thank you for highlighting this critical point. We have carefully reviewed the entire manuscript and revised any language that could imply causality. Terms such as “associated with,” “linked to,” or “potential signal” were used consistently. We have emphasized throughout that FAERS data cannot confirm causation, and this limitation is now more clearly stated in both the Methods and Discussion sections.

Comment 2: Overstatement of Significance for Rare Events

Some ROR/PT values are based on very few cases (e.g., 3 reports for subdural hematoma), and this should be clearly stated.

Response:

We agree. We have added explicit clarification in the Results sections whenever rare events are discussed, noting the small number of cases and urging cautious interpretation. A sentence was added to the limitations section to explain that high RORs from small samples may not be statistically robust.

Comment 3: Clarity and English Language Issues

The manuscript contains awkward phrasing and grammatical errors.

Response:

We appreciate this observation. The manuscript has undergone professional English editing to improve clarity, grammar, and overall readability. We simplified complex sentence structures and ensured the writing is concise and scientifically professional.

Comment 4: Lack of Comparative Context

Comparison with similar biologics like lecanemab would strengthen the paper.

Response:

Thank you. We have included a new paragraph in the Discussion section comparing Aducanumab’s adverse event profile with that of similar anti-amyloid monoclonal antibodies (e.g., lecanemab and gantenerumab), referencing recent FAERS-based studies and publicly available data.

Comment 5: Ethical and Data Transparency Considerations

Ethics statement is marked "N/A", but this should be explicitly stated in the methods section.

Response:

This has been addressed. We now explicitly state in the Methods (“Ethical Considerations”) that ethics approval was not required due to the retrospective use of de-identified public data from FAERS.

We trust that these revisions satisfactorily address all concerns raised by the reviewers and the editor. We sincerely appreciate the opportunity to revise our manuscript and look forward to your further consideration for publication.

Sincerely,

Chuanwei Zhao, on behalf of all authors

Email: zhaochuanwei946@163.com
